# Spatiotemporal Evolution and Drivers of Total Health Expenditure across Mainland China in Recent Years

**DOI:** 10.3390/ijerph20010597

**Published:** 2022-12-29

**Authors:** Junming Li, Xiulan Han

**Affiliations:** School of Statistics, Shanxi University of Finance and Economics, Wucheng Road No. 140, Taiyuan 030006, China

**Keywords:** spatiotemporal evolution, Bayesian statistics, health expenditure, China mainland, Bayesian LASSO model

## Abstract

A substantially growing health expenditure has become an important global issue. Thus, how and why health expenditure is rising should be urgently investigated in systematic research. The Bayesian space-time model and the Bayesian least absolute shrinkage and selection operator (LASSO) model were employed in this study to investigate the spatiotemporal trends and influence patterns of total health expenditure per capita (THEPC) and total health expenditure (THEE) as a share of the gross domestic product (GDP) on the Chinese mainland from 2009 to 2018. The spatial distribution of THEE as a share of GDP in mainland China has shaped a distinct geographical structure with the characteristic of ‘west high/east low’. Its local increasing trends formed a geographical structure that exhibited a ‘north high/south low’ feature. The heterogeneity of the influence patterns of health expenditure was observed from east to west across China. Natural environmental factors, such as air pollution and green coverage, along with changes in dietary structures, have increasingly influenced the growth of health expenditures.

## 1. Introduction

Globally, aggregate health expenditure has increased substantially in the past 20 years in developed and developing countries [1,2,3], and it accounts for more than 10% of the global gross domestic product (GDP) [4]. This trend is even more critical in middle-income countries [5]. Global health expenditure in real terms increased by 3.9% annually from 2000 to 2017, whereas the economy grew by 3.0% [5]. Fostering the sustainable growth of health expenditures has become an important issue for all countries [6]. This situation has also put pressure on policymakers and academics to understand how and why health expenditures are rising [7].

In the populous country of China, health expenditure has also risen quickly in recent years; the annual growth rate of the country’s total health expenditure (THEE) reached 14.71% in 2009–2017, exceeding that of its GDP (11.30%) [8]. Consequently, an increase in THEE as a share of GDP appeared in the same period [8]. Economic development and population distribution in China are unbalanced [9]. Although countrywide growth in THEE is evident in China, the spatial and temporal disparities in the variation in THEE need to be investigated in depth. A deep understanding of the spatiotemporal trends in health expenditures in China can provide a valuable reference for policymakers. To our knowledge, only a few studies have explored the spatiotemporal patterns of health expenditures at the sub-national level in China.

Understanding the main driving factors of health expenditure can help the government to scientifically plan the future of the healthcare system [8]. The determinants or influential factors of health expenditure have been researched in numerous studies, and the influence of economic factors on the THEE per capita (THEPC) has been widely investigated. Researchers have reached a consensus that increasing income can boost health expenditures. As early as 1977, Newhouse pointed out that national income can explain 90% of the variations in THEPC in developed countries [10]. Further, recent studies have concluded that economic growth and financial development significantly increase health expenditure [1,4,11,12,13,14,15,16,17]. Beyond economic factors, the influences of non-economic factors (e.g., population ageing, air pollution, medical technology) have been researched [18,19,20,21]; such studies have mainly focused on pooled sample data of Organization for Economic Co-operation and Development (OECD) countries in the twentieth century [22,23,24,25,26]. Indeed, Martin et al. (2011) [27] reviewed 20 relevant primary studies on the determinants of health expenditure, but they indicated that no single pattern of results could be clearly identified.

Comprehensive evidence on the determinants of health expenditures in China is limited. Hou et al. (2020) [8] explored the factors influencing the Chinese THEPC at the sub-national level based on spatiotemporal panel data across 31 provinces from 2009 to 2016. However, the factors considered in the present study—including four category factors (economic factors, population ageing, epidemiology, and number of beds) and natural factors (e.g., air pollution and climate change)—were not considered in Hou et al.’s study; neither were the interactions of the different factors determined.

In light of the lack of previous research, our study has two targets. First, this study explores the spatiotemporal trends of THEPC and THEE as a share of GDP in recent years in mainland China at the provincial level. Second, the influence patterns of the three categories of factors affecting THEPC and THEE as a share of GDP at the national and subnational levels on the Chinese mainland are investigated.

## 2. Methods

### 2.1. Three Subnational Areas of Chinese Mainland

**Figure 1:**
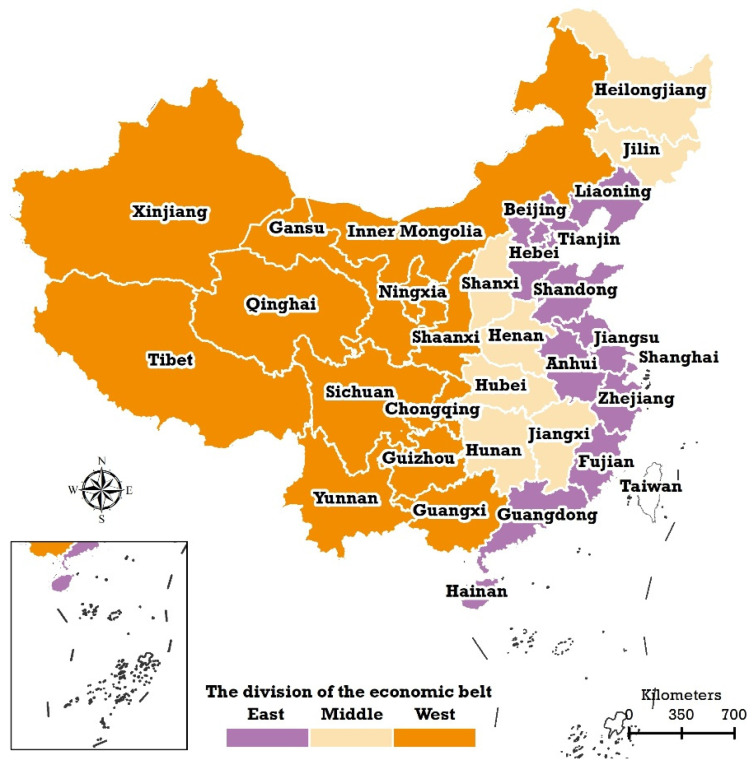
The geographical distributions of the three economical belts of the Chinese mainland: eastern, middle, and western China.

The study area in this paper is the Chinese mainland. Due to the unavailability of data, Taiwan Province, Hongkong, and Macao were not included. On the basis of provincial scale, the Chinese mainland can be divided into three subnational regions: eastern, middle, and western China (Figure 1). The locations of the 31 provinces included in this study are shown in Figure 1. The three subnational areas represent developed, moderately developed, and underdeveloped economic regions, respectively.

### 2.2. Variables Selection and Data Sources

**Figure 2:**
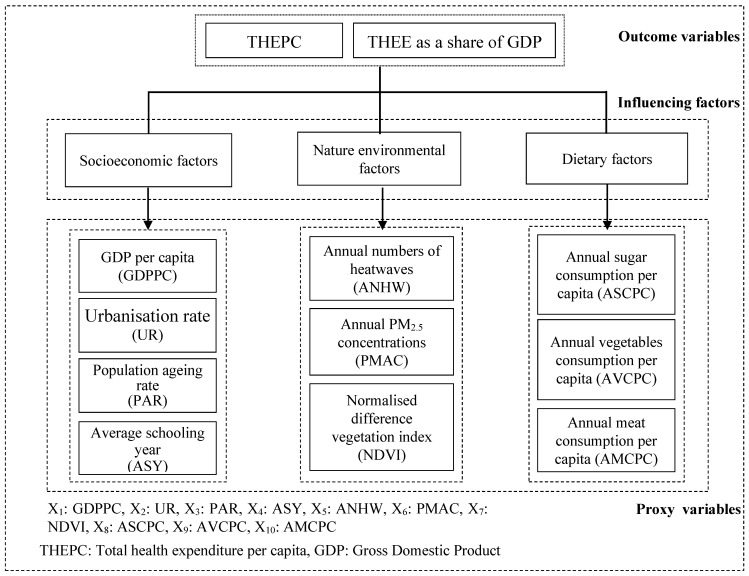
Relationships between outcome variables, the THEPC and the THEE’s share of the GDP, and three categories of influencing variables containing 10 proxy variables.

The data on THEPC (×1000 Chinese yuan) and THEE as a share of the GDP (%) in this study were collected from the China Health Statistical Yearbook (http://www.nhc.gov.cn/mohwsbwstjxxzx/tjtjnj/new_list.shtml, accessed on 16 August 2022), which covered 31 provincial regions of the Chinese mainland in corresponding years. As mentioned above, some studies concluded that economic and social development can significantly accelerate health expenditure [1,4,11,12,13,14,15,16,17]. Hence, we selected four economic factors (Figure 2): GDP per capita (GDPPC), urbanization rate (UR), population ageing rate (PAR), and average schooling years (ASY). Non-economic factors (e.g., air pollution, climate change, dietary structure) may also influence health expenditure [18,19,20,21]; therefore, three nature environmental proxy factors—the annual number of heatwaves (ANHW), annual ${\mathrm{PM}}_{2.5}$ concentrations (PMAC), and the normalized difference vegetation index (NDVI)—and three dietary proxy factors—annual sugar consumption per capita (ASCPC), annual vegetable consumption per capita (AVCPC), and annual meat consumption per capita (AMCPC)—were selected as influencing factors (Figure 2). Data on the socioeconomic factors ASCPC, AVCPC, and AMCPC, were collected from the China Statistical Yearbook (http://www.stats.gov.cn/tjsj/ndsj/, accessed on 16 August 2022). Concerning the environmental factors, data on ANHW were obtained from the following website: https://figshare.com/collections/GHWR_a_multimethod_global_heatwave_and_wawaspell_record_and_toolbox/4004668 (accessed on 16 August 2022) [28]. The data on PMACs, with a spatial resolution of 0.1°×0.1° (~10 km×10 km), were produced by van Donkelaar’s team [29,30]. The data on NDVI were downloaded from the following website: https://www.resdc.cn/data.aspx?DATAID=346 (accessed on 16 August 2022).

### 2.3. Bayesian Spatiotemporal Hierarchy Model

To investigate the spatiotemporal trends of THEPC and THEE as a share of GDP in China, a Bayesian spatiotemporal hierarchical model (BSTHM) [31] was used in this study. The two outcome variables can both be regarded as continuous variables. Although the initial value of the THEE share of GDP is at (0, 1), we modelled its percentage multiplied by 100. Consequently, the likelihood distributions of the two dependent variables were all assigned a log-normal distribution, expressed as follows:

(1) $$\mathrm{THEPC}:\;Y_{it}\sim LogN\left(\mu_{it},\sigma^{2}\right)$$

(2) $$\mathrm{THEE}\;\mathrm{as}\;a\;\mathrm{share}\;\mathrm{of}\;\mathrm{GDP}:\;y_{it}\sim LogN\left(\gamma_{it},\partial^{2}\right)$$

where $Y_{it}$ and $y_{it}$ represent THEPC and THEE as a share of GDP of the *i*-th provincial region in the t-th year; $\mu_{it}$ and $\gamma_{it}$ are the corresponding expectancy values; and $\sigma^{2}$ and $\partial^{2}$ are the corresponding variances, whose priors were assigned with Gamma distributions. The spatiotemporal process model is expressed as follows:

(3) $$\mathrm{THEPC}:\;\exp(\mu_{it}+\frac{\sigma^{2}}{2})=\alpha^{\left(r\right)}+S_{i}^{\left(r\right)}+\left(\beta_{0}^{\left(r\right)}t^{*}+v_{t}^{\left(r\right)}\right)+\beta_{1i}^{\left(r\right)}t^{*}+\varepsilon_{it}^{\left(r\right)}$$

(4) $$SRM_{i}^{\left(r\right)}=\frac{\alpha^{\left(r\right)}+S_{i}^{\left(r\right)}}{\alpha^{\left(r\right)}}$$

(5) $$\mathrm{THEE}\;\mathrm{as}\;a\;\mathrm{share}\;\mathrm{of}\;\mathrm{GDP}:\;\;\exp\left(\gamma_{it}+\frac{\partial^{2}}{2}\right)=\alpha^{\left(p\right)}+S_{i}^{\left(p\right)}+\left(\beta_{0}^{\left(p\right)}t^{*}+v_{t}^{\left(p\right)}\right)+\beta_{1i}^{\left(p\right)}t^{*}+\varepsilon_{it}^{\left(p\right)}$$

(6) $$SRM_{i}^{\left(p\right)}=\frac{\alpha^{\left(p\right)}+S_{i}^{\left(p\right)}}{\alpha^{\left(p\right)}}\;$$

(7) $$t^{*}=\frac{1+T}{2}$$

where $t^{*}$ and *T* represent the middle and length of the study period, respectively; *T* is 10 in this paper. $\alpha^{\left(r\right)}$ and $\alpha^{\left(P\right)}$ are the basic fixed constants for THEPC and THEE as a share of GDP, respectively; their corresponding priors used non-informative prior distributions. $SRM^{\left(r\right)}$ and $SRM^{\left(p\right)}$ represent the spatial relative magnitudes (*SRM*) of THEPC and THEE as a share of GDP, comparing them to the national overall baseline level. $\left(\beta_{0}^{\left(r\right)}t+v_{t}^{\left(r\right)}\right)$ and $\left(\beta_{0}^{\left(p\right)}t+v_{t}^{\left(p\right)}\right)$ describe the general trend of the Chinese THEPC and THEE as a share of GDP in 2009–2018. The priors of $\beta_{0}^{\left(r\right)}$ and $\beta_{0}^{\left(p\right)}$ were assigned non-informative prior distributions. The priors of $v_{t}^{\left(r\right)}$ and $v_{t}^{\left(p\right)}$ were assigned with Gaussian distribution with zero mean; $\beta_{1i}^{\left(r\right)}$ and $\beta_{1i}^{\left(p\right)}$ represent the local trends of THEPC and THEE as a share of GDP in the *i*-th provincial region during 2009–2018. Prior distributions of the parameters, $SRM_{i}^{\left(r\right)}$, $SRM_{i}^{\left(p\right)}$, $S_{i}^{\left(r\right)}$, $S_{i}^{\left(p\right)}$, $\beta_{1i}^{\left(r\right)}$, and $\beta_{1i}^{\left(p\right)}$ were assigned using the Besag York Mollie (BYM) model [32], expressing the spatial structured and unstructured random effects. The spatial adjacency matrix adopts the first-order ‘Queen’ adjoining form; $\varepsilon_{it}^{\left(r\right)}$ and $\varepsilon_{it}^{\left(p\right)}$ represent the Gaussian random terms, and their priors were assigned with Gaussian distribution with zero mean [33].

### 2.4. Bayesian LASSO Regression Model

The Bayesian LASSO (B-LASSO) model [34] was adopted in our study to overcome the problem of multicollinearity among the variables. The B-LASSO model is the Bayesian version of the ordinary LASSO regression model [35]. In the B-LASSO model, the likelihood distributions of THEPC and THEE as a share of GDP were also all assigned to the log-normal distribution, and the regression parameters were assigned to the independent and identical Laplace priors. Bayesian LASSO estimations differ from ordinary least squares (OLS), penalized by the least squares method, which minimizes the residual sum of squares while controlling the L1 norm of the coefficient vector of regression [34,35]. The B-LASSO model can acquire a more stable estimation and automatically provide interval estimates for all parameters, including the error variance [34]. The B-LASSO model of the relationship among the influence factors, $x_{j}\;\left(j=1,\ldots ,n\right),$ n is the number of the influence factors, set as 10 in this paper, and the outcome variables THEPC and THEE as a share of GDP. The mathematical structure can be expressed as follows:

(8) $$\mathrm{THE}:\;\exp(\mu_{i,t}+\frac{\sigma^{2}}{2})=\varphi^{\left(r\right)}+s_{i}^{\left(r\right)}+v_{t}^{\left(r\right)}+\sum_{j=1}^{n}\beta_{j}^{\left(r\right)}x_{j,i,t}+\xi_{it}^{\left(r\right)}$$

(9) $$\mathrm{THEE}\;\mathrm{as}\;a\;\mathrm{share}\;\mathrm{of}\;\mathrm{GDP}:\;\exp(\gamma_{it}+\frac{\partial^{2}}{2})=\varphi^{\left(p\right)}+s_{i}^{\left(p\right)}+v_{t}^{\left(p\right)}+\sum_{j=1}^{n}\beta_{j}^{\left(p\right)}x_{j,i,t}+\xi_{it}^{\left(p\right)}$$

(10) $$\hat{\beta }=argmin_{\beta }{\left(\hat{Y}-\beta X\right)}^{T}\left(\hat{Y}-\beta X\right)+\tau {\left\|\beta \right\|}_{1}$$

(11) $$\beta |\tau ,\sigma_{\beta }^{2}\sim \overset{n}{\underset{j=1}{\prod }}\frac{\tau }{2\sigma_{\beta }}e^{-\frac{\tau \left|\beta_{j}\right|}{\sigma_{\beta }}}$$

where $\;\varphi^{\left(r\right)}$ and $\varphi^{\left(p\right)}$ are overall intercepts whose priors were assigned to non-informative priors. $s_{i}^{\left(r\right)}$ and $s_{i}^{\left(p\right)}$, $v_{t}^{\left(r\right)}$, and $v_{t}^{\left(p\right)}$ represent the spatial and temporal fixed effects. The priors of $s_{i}^{\left(r\right)}$ and $s_{i}^{\left(p\right)}$ were also assigned by the BYM model. Gaussian distribution with zero mean was assigned to $v_{t}^{\left(r\right)}$, and $v_{t}^{\left(p\right)}$. $\beta_{j}^{\left(r\right)}$, and $\beta_{j}^{\left(p\right)}$ are the B-LASSO regression parameters of the influence variables $x_{jit}$; n is the number of the influence variables; $\xi_{it}^{\left(r\right)}$ and $\xi_{it}^{\left(p\right)}$ represent the random error whose priors were assigned by Gaussian distribution with zero mean. $\hat{Y}$ and $\hat{\beta }$ represent the estimate of the dependent variables, Y, and regression parameters, β. X is the matrices of $x_{j,i,t}$. $\sigma_{\beta }^{2}$ represents posterior variance of the regression parameters, β. The coefficient τ is greater than 0 and determines the amount of shrinkage.

These Bayesian statistical estimations were implemented using WinBUGS 14 software [36]. The convergence of all the Bayesian estimations was assessed using the Gelman–Rubin statistical coefficient [37], where the closer the value is to 1.0, the better the convergence is. The Gelman–Rubin statistical values of all model parameters ranged from 0.96 to 1.05.

### 2.5. GeoDetector Model

The GeoDetector model [38,39] can detect the explanatory power of individual variable The primary idea of the GeoDetector model is that two variables (linearly or non-linearly) are accordant in strata if one causes or affects the other. The GeoDetector model may capture non-linear association effects, making it different from the ordinary linear regression model. A q-statistic value produced by the GeoDetector model can quantify the explanatory power of a single factor. The q-statistic value can be calculated as follows:

(12) $$q_{x_{j}}=(1-\frac{\sum_{h=1}^{l}N_{j,h}\sigma_{j,h}^{2}}{N\sigma^{2}})\times 100\%$$

where *h* (*h* = 1,2, …, *l*) represents the strata of a single variate $X_{j}$, $N_{j,h}$ and *N* are the numbers of units in the stratum h of $X_{j}$ and in the total regions, respectively. In addition, $\sigma_{j,h}^{2}$ and $\sigma^{2}$ represent the variances of the THEPC and the THEE’s GDP share in the stratum *h* of $X_{j}$ and in 31 provincial regions of Chinese mainland in 2009–2018, respectively. The magnitude of the q-statistic value, $q_{x_{j}}$, quantifies the explanatory power of $X_{j}$. The range of the q-statistic value is from 0% to 100%, and the larger the q-statistic value, the greater its explanatory power on the THEPC and the THEE’s GDP share. The significance of the q-statistic value was tested by the noncentral F-test.

## 3. Results

### 3.1. Descriptive Statistics

**Figure 3:**
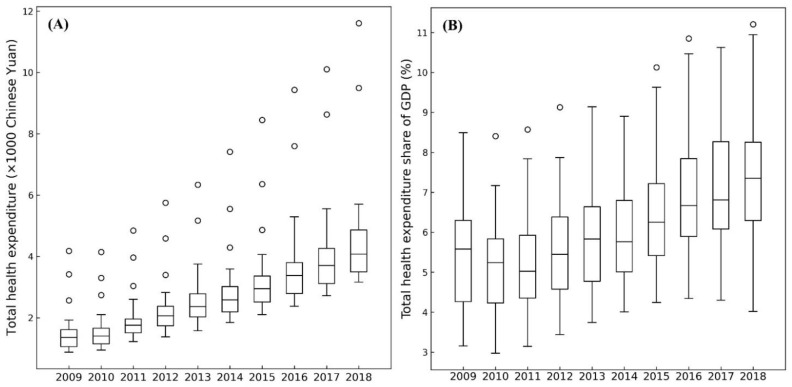
Boxplot of Chinese total health expenditure per capita (**A**) and total health expenditure as a share of GDP (**B**) from 2009 to 2018.

Health expenditures in China showed a trend of continuous growth in our analysis. THEPC and THEE as a share of GDP throughout China increased from 1314.26 Chinese yuan and 5.08% in 2009 to 4236.98 Chinese yuan and 6.43% in 2018. THE’s annual growth rate was 14.89%. The annual growth of THEE as a share of GDP was 0.19%. Moreover, the dispersity of the various regions was increasingly obvious, especially for THEPC. Figure 3 illustrates the boxplots of THEPC and THEE as a share of GDP at the provincial level in China from 2009 to 2018. This reveals that not only did the significant increasing trends of the two terms of health expenditure occur throughout China, but the differences between various provincial regions also grew overall. The maximum ranges of THEPC and THEE as a share of GDP increased from 3304.78 Chinese yuan and 5.34% in 2009 to 8449.36 Chinese yuan and 7.19% in 2018.

**Figure 4:**
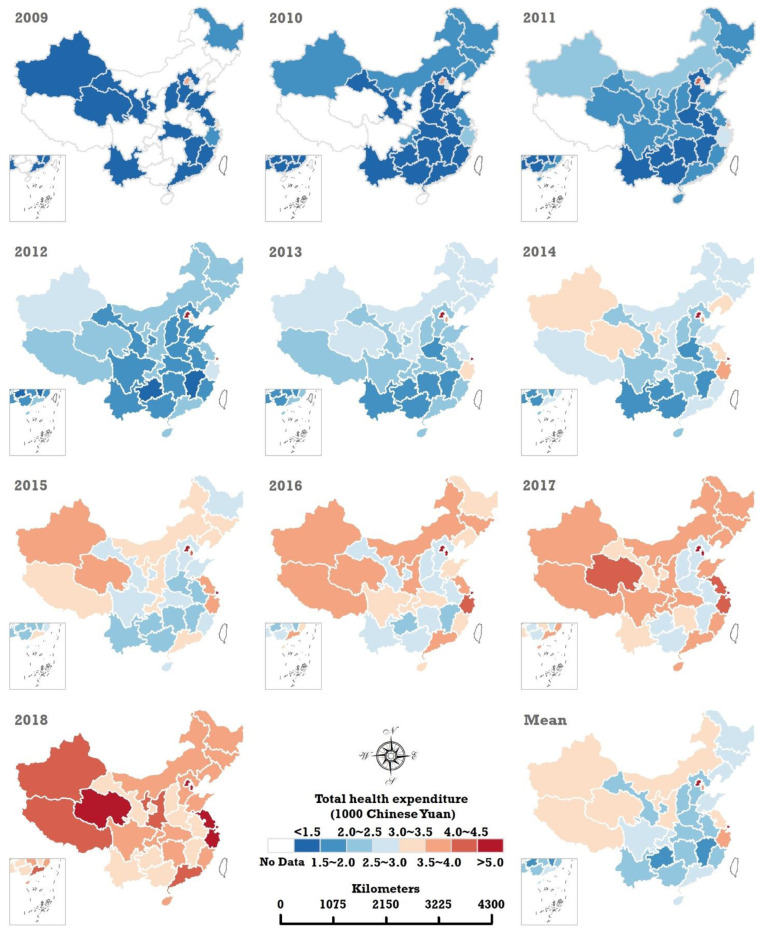
Spatiotemporal sequence map and spatial pattern of the mean THEE in China from 2009 to 2018.

**Figure 5:**
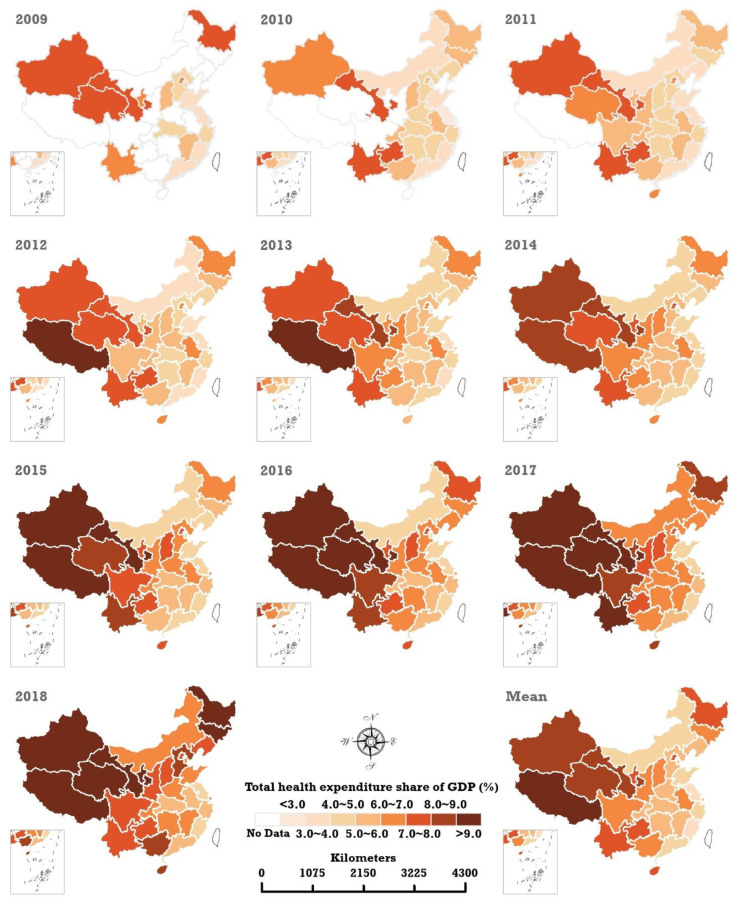
Spatiotemporal sequence map and spatial pattern of the mean THEE as a share of GDP in China from 2009 to 2018.

Figure 4 and Figure 5 illustrate the spatiotemporal sequence map and spatial pattern of the mean of THEPC and THEE as a share of GDP in China from 2009 to 2018. They show that THEPC and THEE as a share of GDP in each provincial region increased substantially in the decade.

During the study period, the top three THEPC results always emerged in the three cities directly under the central government—Beijing, Shanghai, and Tianjin. The THEPC of Beijing has exceeded 10,000 Chinese yuan since 2017, reaching 11,609.06 Chinese yuan in 2018. THEPC in Shanghai and Tianjin were 9495.89 and 5698.41 Chinese yuan, respectively, in 2018. The list of cities with the bottom three THEPC changed from Yunnan (935.19 Chinese yuan), Jiangxi (886.51 Chinese yuan), and Guizhou (875.00 Chinese yuan) in 2009 to Henan (3227.66 Chinese yuan), Jiangxi (3170.63 Chinese yuan), and Anhui (3159.72 Chinese yuan) in 2018. 

An obvious increasing trend of THEE as a share of GDP also occurred in each of the 31 provincial regions in China. A distinct spatial geographical structure of THEE as a share of GDP has emerged since 2016. Interestingly, the top three THEE as a share of GDP occurred in three provincial regions in western China in 2009, namely, Tibet (8.49%), Gansu (7.79%), and Xinjiang (7.74%). By 2019, three western provinces—Qinghai (11.21%), Tibet (10.84%), and Heilongjiang (10.95%)—became the top three areas with the highest THEE as a share of GDP. The lowest THEE as a share of GDP occurred in the eastern regions of China. In 2009 and 2018, the bottom three regions with the lowest THEE share of the GDP were always Jiangsu (3.15% in 2009 and 4.33% in 2018), Fujian (3.23% in 2009 and 4.02% in 2018), and Guangdong (3.30% in 2009 and 5.20% in 2018).

### 3.2. Steady Spatial Patterns

**Figure 6:**
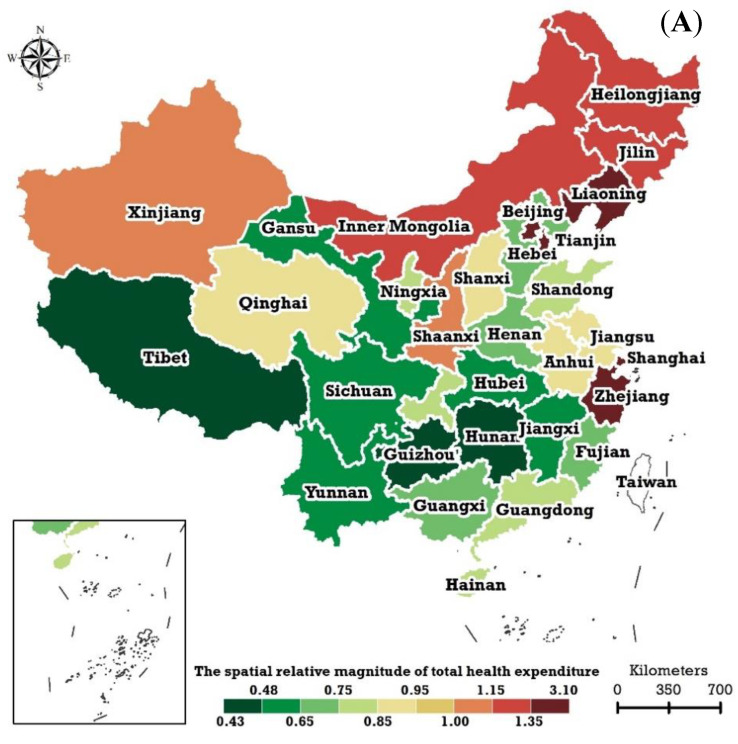

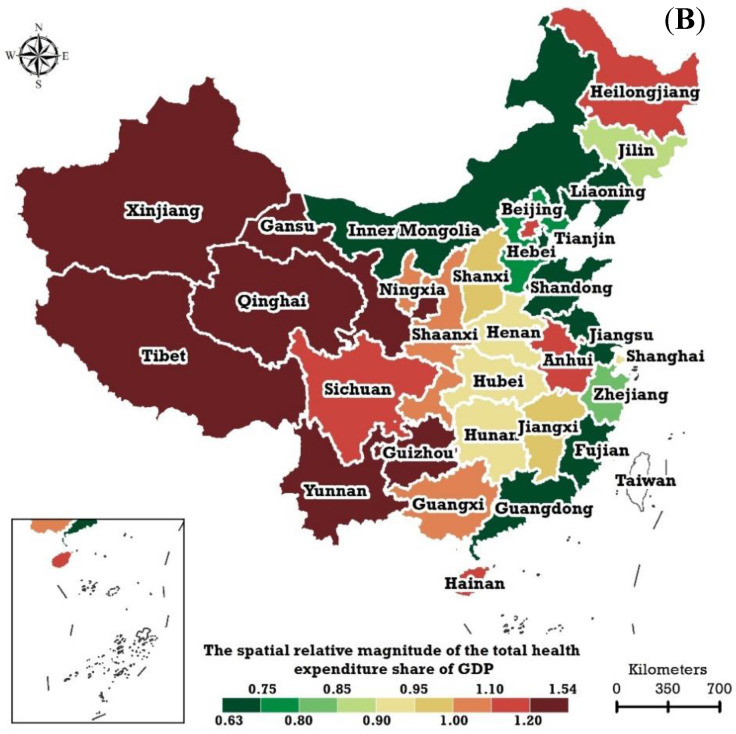
Spatial relative magnitude of (**A**) total health expenditure per capita and (**B**) total health expenditure as a share of GDP in China, estimated by the Bayesian spatiotemporal hierarchy model.

The steady spatial pattern, quantified with the SRM to the nationwide overall level estimated by the BSTHM, was explored in this study. Figure 6A shows the SRM of THEPC in China. The SRM of THEPC did not form a particularly obvious geographical structure. The SRM with values significantly greater than 1.00 emerged in the eight provincial regions of Beijing (3.10 [95% confidence interval (95% CI): 2.37, 4.13]), Shanghai (2.54 [95% CI: 1.98, 3.41]), Tianjin (2.13 [95% CI: 1.73, 2.72]), Zhejiang (1.45 [95% CI: 1.25, 1.75]), Liaoning (1.35 [95% CI: 1.14, 1.64]), Inner Mongolia (1.33 [95% CI: 1.15, 1.68]), Heilongjiang (1.27 [95% CI: 1.13, 1.48]), and Jilin (1.19 [95% CI: 0.99, 1.45]). SRMs with values significantly less than 1.00 occurred in 21 provincial regions, the bottom five of which were Guizhou (0.43 [95% CI: 0.12, 0.64]), Tibet (0.46 [95% CI: 0.28, 0.85]), Hunan (0.48 [95% CI: 0.16, 0.69]), Sichuan (0.52 [95% CI: 0.14, 0.74]), and Jiangxi (0.54 [95% CI: 0.30, 0.71]). The SRMs of two regions—Shaanxi and Xinjiang—were equivalent to the overall nationwide level.

Figure 6B exhibits the SRM of THEE as a share of GDP in China. The SRMs showed a distinct spatial structure, with a ‘west high/east low’ feature. The nine provincial regions located in western China—Tibet (1.54 [95% CI: 1.35, 1.75]), Gansu (1.38 [95% CI: 1.26, 1.52]), Xinjiang (1.37 [95% CI: 1.25, 1.49]), Yunnan (1.36 [95% CI: 1.25, 1.49]), Guizhou (1.35 [95% CI: 1.23, 1.49]), Qinghai (1.28 [95% CI: 1.15, 1.42]), Anhui (1.17 [95% CI: 1.07, 1.29]), Beijing (1.15 [95% CI: 1.04, 1.28]), and Heilongjiang (1.12 [95% CI: 1.01, 1.22])—had SRMs of THEE as a share of GDP that were significantly greater than 1.00. The eight provincial regions located in eastern China—Shanghai (0.90 [95% CI: 0.80, 1.00]), Liaoning (0.68 [95% CI: 0.57, 0.78]), Zhejiang (0.85 [95% CI: 0.77, 0.93]), Guangdong (0.65 [95% CI: 0.57, 0.73]), Tianjin (0.73 [95% CI: 0.65, 0.81]), Shandong (0.70 [95% CI: 0.58, 0.74]), Jiangsu (0.63 [95% CI: 0.53, 0.70]), and Fujian (0.67 [95% CI: 0.58, 0.75])—had SRMs of THEE as a share of GDP that were significantly less than 1.00. Nine provincial regions (Henan, Jiangxi, Shanxi, Guangxi, Chongqing, Ningxia, Shaanxi, Hainan, and Sichuan) possessed a level of THEE as a share of GDP that was parallel to the national overall level.

### 3.3. Local Temporal Trends

**Figure 7:**
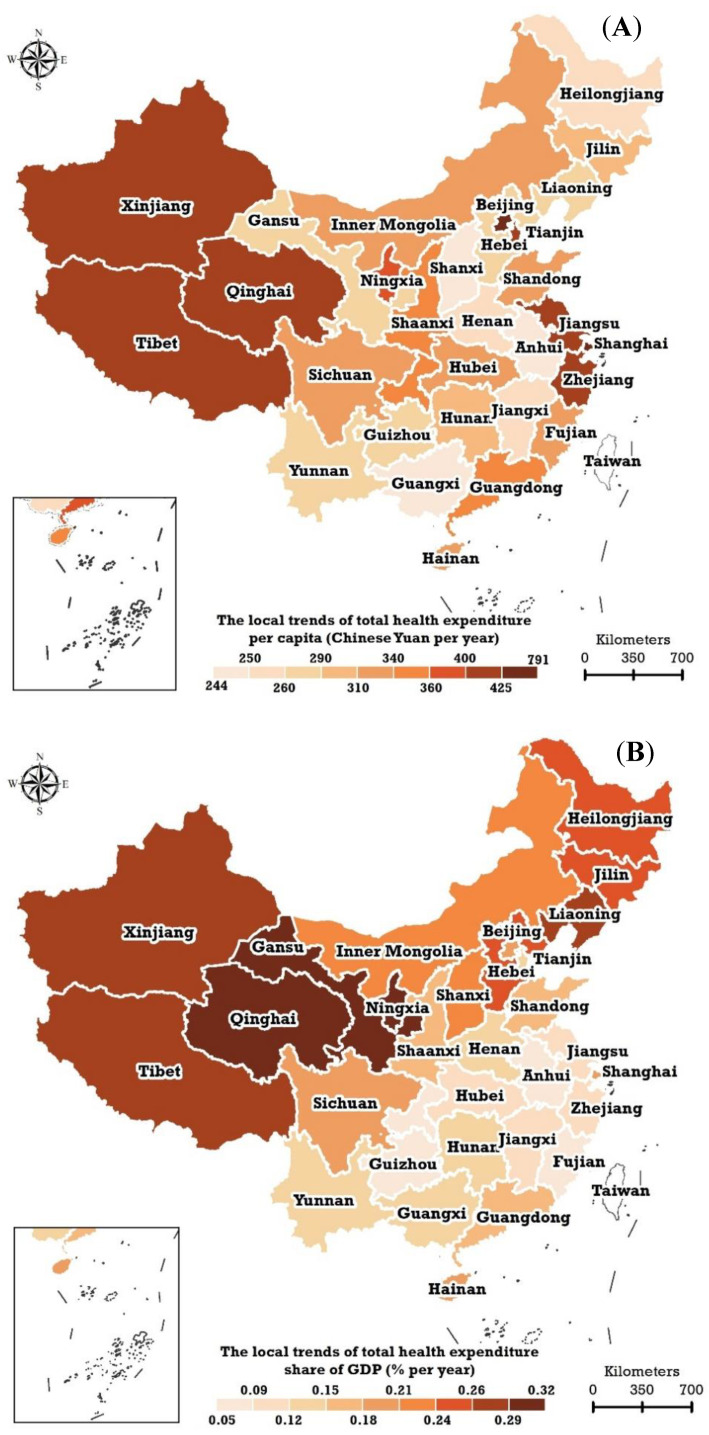
Local trends of (**A**) total health expenditure per capita Chinese yuan per year) and (**B**) total health expenditure as a share of GDP (% per year) in China estimated using the Bayesian spatiotemporal hierarchy model.

This study used the BSTHM to estimate the local trends of THEPC (Figure 7A) and THEE as a share of GDP (Figure 7B) in China from 2009 to 2018, based on extracting stable spatial patterns. The THEPCs in the 31 studied provincial regions all increased during 2009–2018. The top five regions with the highest local trends of THEPC were Beijing, Shanghai, Jiangsu, Qinghai, and Tibet. The corresponding estimated annual trends were 791 (95% CI: 696, 882), 637 (95% CI: 547, 714), 424 (95% CI: 350, 485), 417 (95% CI: 334, 475), and 412 (95% CI: 327, 494) Chinese yuan per year, respectively. The bottom five regions with the lower local trends of THEPC were Guangxi, Anhui, Shanxi, and Jiangxi all of which are located in central China. The local trends of THEPC in the remaining 21 provincial regions covered a range of 259 to 407 Chinese yuan per year.

The local trends of THEE as a share of GDP throughout China during 2009–2018 demonstrated a conspicuous geographical structure with a ‘north high/south low’ feature. The nine regions located in northern China (dark red in Figure 7B), such as Qinghai (0.32% [95% CI: 0.17%, 0.46%]), Gansu (0.31% [95% CI: 0.16%, 0.46%]), and Ningxia (0.30% [95% CI: 0.15%, 0.46%]) possessed annual trends of greater than 0.25%. The bottom three local trends of THEE as a share of GDP were evident in Anhui, Guizhou, and Chongqing; the corresponding values were 0.05% (95% CI: −0.08%, 0.19%), 0.07% (95% CI: −0.08%, 0.21%), and 0.07% (95% CI: −0.07%, 0.22%) per year, respectively.

### 3.4. Influence Pattern

#### 3.4.1. National Influence Pattern

**Figure 8:**
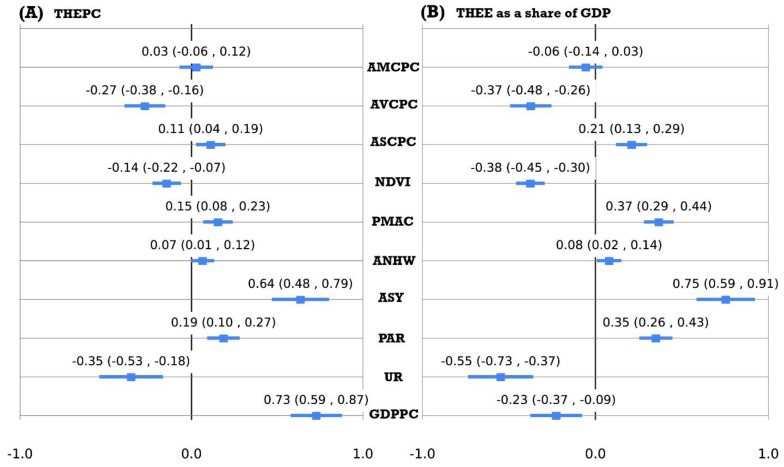
Forest plotting and estimations of the normalized Bayesian LASSO regression coefficients between the 10 independent variables: GDPPC, UR, PAR, ANHW, PMAC, NDVI, ASCPC, AVCPC, AMCPC, and THEPC (**A**) and THEE as a share of GDP (**B**).

The influence factors and their influential effects on THEPC and THEE as a share of GDP in Chinese mainland at the national level were estimated using the B-LASSO model. Nationally, nine factors—GDPPC, UR, PAR, ASY, PMAC, ANHW, NDVI, ASCPC, and AVCPC—significantly affected THEPC and THEE as a share of GDP (Figure 8). Specifically, the top three dominant influencing factors affecting THEPC are GDPPC, ASY, and UR; the corresponding influence contributions were 27.5%, 24.2%, and 13.2%, respectively. However, the influence pattern of THEE as a share of GDP was different from that of THEPC. The two main influencing factors are ASY and UR, the corresponding influence attributions were 22.8% and 16.7%, and those of three factors, NDVI, AVCPC, and PMAC, were parallel: 11.6%, 11.2%, and 11.2%, respectively. AMCPC did not significantly influence THEPC and THEE as a share of GDP.

Furthermore, the specific influence effects of the significant factors on THEPC and THEE as a share of GDP at the national level were estimated by the B-LASSO model and are listed in Table 1. The results show that the increase of six factors—GDPPC, PAR, ASY, ANHW, PMAC, and ASCPC—may lead to an increase in THEPC. The concrete increments of THEPC can be found in Table 1, when the six factors, GDPPC, PAR, ASY, ANHW, PMAC, and ASCPC, increased by 1000 Chinese yuan, one percent, one year, one day, one microgram per stere, and one kilogram, respectively, if other variables were invariant. Inversely, an increase in the other three factors, UR, NDVI, and AVCPC, may contribute to a decrease in THEPC. The corresponding decrements of THEPC are listed in Table 1.

Except for GDPPC, the other nine factors maintained the same relationship between THEE as a share of GDP and THEPC (Table 1). The estimations showed that it may lead to an increase of THEE as a share of GDP when the five factors PAR, ASY, ANHW, PMAC, and ASCPC increased. Conversely, increase of the four factors GDPPC, UR, NDVI, and AVCPC may affect a decrease of THEE as a share of GDP. The corresponding concrete increments and decrements of THEE as a share of GDP are listed in Table 1.

#### 3.4.2. Model Validation

To validate the results estimated from the B-LASSO model, the GeoDetector model was employed to detect the national influence pattern. As previously mentioned, the GeoDetector model can estimate the explanatory powers of the 10 factors on the THEPC and the THEE’s GDP share. Table 2 lists the specific results. It should be pointed out that the estimated values of the explanatory powers by the GeoDetector model is non-negative. Compared with the results of the national influence pattern estimated from the B-LASSO model (Figure 8), it can be seen that the explanatory powers of the selected 10 factors are almost equivalent to the absolute normalized regression coefficients estimated by the B-LASSO model. This indicates that the results of the B-LASSO model are robust.

The ordinary least squares (OLS) regression model was also used to validate the estimated results by the B-LASSO model. The corresponding mathematic expression is as follows:

(13) $$y_{it}=\alpha +S_{i}+T_{t}+\sum_{j=1}^{10}\beta_{j}X_{j}+\varepsilon_{it}$$

where $y_{it}$ represents THEPC or THEE’s GDP share; α is intercept; $S_{i}$ and $T_{t}$ represent spatial and temporal fixed effect; $\beta_{j}$ and $X_{j}$ represent the j-th OLS regression coefficient and influencing factor; $\varepsilon_{it}$ is error term.

The estimated results of the OLS regression coefficients list in the Table 3. Generally, the influencing directions of the 10 variables is in accordance with the estimations of the B-LASSO model. Furthermore, it can be seen that the regression coefficients values of the OLS regression model are not significantly different from that of the B-LASSO model. The maximal relative differences of all the regression coefficients are less than 15%, some are even very close. This validation demonstrates again the robustness of the B-LASSO model’s estimation.

#### 3.4.3. Subnational Influence Pattern

To consider the spatial heterogeneity, the influence patterns of THEPC and THEE as a share of GDP at the subnational level of three economic areas—east, middle, and west (Figure 1)—were also explored in this study. As previously mentioned, the three subnational areas represent three different levels of the economic development: developed, moderately developed, and underdeveloped, respectively.

Table 4 lists the B-LASSO model’s estimations of the influential effects of the independent variables affecting THEPC in the three subnational areas of Chinese mainland. The results showed that the influencing patterns of the three subnational areas are different. GDPPC significantly and positively affected THEPC in the three subnational regions, and the corresponding influence effects gradually increased from the east, middle, to the west. PAR was a significant influence factor on THEPC in the middle and west; however, this influence was not significant in the east. The ASY significantly affected THEPC only in the east. The environmental factor PMAC significantly and positively affected THEPC in the east and middle but not significantly in the west. The dietary factor, AVCPC, negatively affected THEPC in the east, but this factor was not significantly associated with THEPC in the middle and west. The natural factor, NDVI, was a significant influence factor only in the west. Other factors, UR, ANHW, ASCPC, and AMCPC, were not significantly influential factors in the three subnational regions.

The results of the B-LASSO’s regression coefficients of the 10 influence factors on THEE as a share of GDP (%) at subnational scale, east, middle, and west of the Chinese mainland are listed in Table 5. Similarly, the heterogeneity of the influence patterns of the 10 factors on THEE as a share of GDP existed in the three subnational regions. For socioeconomic factors, ASY was associated significantly with THEE as a share of GDP in the east, PAR was significant in the middle, and UR and PAR were significant in the west. In terms of natural environmental factors, ANHW and NDVI influenced significantly THEE as a share of GDP only in the west, whereas PMAC influenced significantly THEE as a share of GDP in the east and middle. The different dietary factors had different influence effects in the three subnational regions. An increase of one kilogram of ASCPC and AVCPC in the east may lead to THEE as a share of GDP decreasing by 1.159% (95% CI: 0.333%, 2.913%) and 0.040% (95% CI: 0.005%, 0.074%), respectively. AVCPC and AMCPC correlated negatively with THEE as a share of GDP in the middle and the west, respectively.

## 4. Discussion

The spatiotemporal trends and influencing patterns of THEPC and THEE as a share of GDP in China from 2009 to 2018 were explored in this study. The BSTHM and B-LASSO models were employed to explore these two terms. The two outcome variables represent health expenditure from absolute and relative angles.

Generally, China’s THEPC is much less than that of the global average and developed countries, for example, the United States and Germany, according to data from the World Bank (https://data.worldbank.org/indicator/, accessed on 16 August 2022). China’s THEE as a share of GDP is of an above-average level; however, it is still below that of Western nations, such as the United Kingdom and the United States. The sustainable increasing trend of THEE as a share of GDP in China is different from the global trend, which has shown a decreasing trend since 2016.

Although THEPC and THEE as a share of GDP at the provincial level throughout China both increased from 2009 to 2018, the spatial diversities of both of the rising tendencies are distinct. The steady geographical structures of the SRMs and local trends of THEE as a share of the GDP are more apparent than those of THEPC. We argue that the relative index—THEE as a share of the GDP—has less uncertainty compared with the absolute index—THEPC. We assume that THEE as a share of GDP in the regions in western China is higher because these areas are underdeveloped, and the corresponding GDP is not yet high. Consequently, the THEPC of the developed provincial regions was higher than that of the undeveloped areas. Although the THEPC in western provincial areas are not high, the local areas show increasing trends. Surprisingly, not only is THEE as a share of the GDP in the western provincial regions higher than the overall nationwide level but the corresponding local trends are also higher.

Nationally, the increase in PAR and ASY may contribute to an increase in health expenditures. The results showed that climate change (heatwave) and air pollution should increase health expenditure; however, an increase in vegetative cover may reduce health expenditures. This discovery can provide evidence for policy making to reduce health expenditure. The statistical results also provided information that a decrease in sugar intake and an increase in vegetable intake may lower health expenditures. From the perspective of regional heterogeneity, the influence patterns of health expenditure exhibited differences on the subnational scale. The residents’ education level affects health expenditure in the east, but not in the middle or the west. We assume that the baseline education level of the east is higher than that of the middle and the west; therefore, the residents’ education level’s sensibility to health expenditure in the east is consequently higher. The influential effect of the heat wave is not detected at the subnational scale, except for THEE as a share of GDP in the west. Air pollution significantly impacted health expenditure in the east and middle but not in the west. One possible reason is that the pollution level of the west is lower than that of the east and the middle, and consequently the influencing effects of ${\mathrm{PM}}_{2.5}$ in health expenditures is not significant in the west. Some researchers have concluded that exposure to air pollution is associated with an increase in health expenditure, especially ${\mathrm{PM}}_{2.5}$ pollution [39]. Some scholars have also pointed out that the growth of ${\mathrm{CO}}_{2}$ and Nitrous oxide emissions can increase health expenditure [40]. According to the results of our study and the previous research, air pollution and climate change are the major negative factors for an increase of health expenditure. It is interesting that the influence of the effect of NDVI on health expenditures only occurred in the west. A known fact is that the vegetation cover of the west is lower than that of the middle and the east. Hence, an increase in the NDVI can reduce health expenditures. Dietary factors have a more significant influence on THEE as a share of GDP, although the specific factor is not the same in the three areas. The dietary structure needs to adjust in the east and the middle, but nutrition needs to improve in the west.

In general, spatial and temporal heterogeneity should be considered during the process of making relevant policies about decreasing health expenditures in China and other countries around the world. The results of the influence patterns of THEPC and THEE as a share of GDP can also provide some revelations for predicting and controlling health expenditures in a precise manner. Environmental factors, such as ${\mathrm{PM}}_{2.5}$ pollution and green coverage, have been growing increasingly important for the rise in health expenditure. The results of this study indicate that the decrease of ${\mathrm{PM}}_{2.5}$ concentrations can reduce THEE in eastern and middle China; nevertheless, the increase in green cover can reduce the THEE in the western China. Simultaneously, a change in dietary structure is also a vital factor influencing health expenditures across China. Besides environmental factors, improving access to healthcare may also reduce THEE. Easier healthcare accessibility can enable patients, especially the elderly and children, to receive timely medical care [41,42,43,44] and then reduce medical expenses. Therefore, it is important for decreasing the THEE of China to maximize the effectiveness of the healthcare system. This suggestion works elsewhere, too. Although we hope that this study has deeply and comprehensively investigated the problem of health expenditure in China as far as possible, there are some limitations to this study. First, the provincial area was taken as the spatial statistical unit in our study, but this delineation was not sufficiently fine in terms of spatial granularity. If the spatial units were to be further subdivided, the problem could be studied in greater detail. Second, this study only discussed the factors associated with THEPC and THEE as a share of GDP; it did not investigate the causal mechanisms. Future studies should consider this aspect by employing causal inference methods. It should also be noted that it is interesting to see how COVID-19 affects health expenditure in mainland China. Unfortunately, the related data covering any period of COVID-19 in China are not available. However, this is a potential topic to be addressed in the future if the relevant data can be collected.

## 5. Conclusions

Based on our findings, this study offers some conclusions. First, the spatial distribution of THEPC in China did not form a particularly obvious geographical structure, whereas the THEE as a share of GDP in China displayed a distinct spatial structure, with a ‘west high/east low’ feature. Second, THEPC and THEE as a share of GDP in the 31 studied provincial regions of China all increased during 2009–2018, and the local trends of THEE as a share of GDP throughout China demonstrated a geographical structure with a ‘north high/south low’ feature. Third, the influence patterns of THEPC and THEE as a share of GDP are distinct. The dominant influencing factors of THEPC are the degree of development, represented by GDPPC and ASY; the dominant influencing factors of THEE as a share of GDP are ASY and UR. Fourth, the heterogeneity of the influence patterns of health expenditures exists from the east to the west in Chinese mainland. Fifth, natural environmental factors, such as air pollution and green coverage have been growing increasingly important for the rise of health expenditure; simultaneously, the change of dietary structure is also a vital factor influencing health expenditure.

## Figures and Tables

**Table 1:** The influence factors’ effects on THEPC and THEE as a share of GDP at the national level, estimated by the B-LASSO model.

	THEPC (Chinese Yuan)			THEE as a Share of GDP (%)		
	Mean	2.5%th	97.5%th	Mean	2.5%th	97.5%th
GDPPC	45.096 **	27.937	61.635	−0.016 **	−0.034	−0.003
UR	−39.488 **	−77.518	−10.897	−0.069 **	−0.112	−0.025
PAR	134.745 **	15.132	255.799	0.281 **	0.140	0.413
ASY	840.935 **	448.234	1256.113	1.114 **	0.672	1.599
ANHW	30.102 *	8.914	84.286	0.041 *	0.014	0.100
PMAC	12.624 **	2.459	25.085	0.034 **	0.002	0.047
NDVI	−127.500 **	−259.396	−5.276	−0.037 **	−0.051	−0.023
ASCPC	324.994 *	57.561	645.744	0.678 **	0.172	1.162
AVCPC	−22.508 **	−39.886	−4.965	−0.035 **	−0.054	−0.015
AMCPC	6.186	−34.025	42.200	−0.014	−0.055	0.030

Footnote: the single * represents a significance of 0.05; the double ** represents a significance of 0.01.

**Table 2:** The estimated explanatory powers of the selected 10 variables on the THEPC and the THEE’s GDP share by the GeoDetector model.

	GDPPC	UR	PAR	ASY	ANHW	PMAC	NDVI	ASCPC	AVCPC	AMCPC
THEPC	0.71 **	0.37 **	0.18 **	0.65 **	0.07 *	0.16 **	0.15 **	0.10 *	0.28 **	0.04
THEE as a share of GDP	0.25 **	0.58 **	0.36 **	0.74 **	0.08 *	0.38 **	0.39 **	0.22 **	0.37 **	0.05

Footnote: the single * represents a significance of 0.05; the double ** represents a significance of 0.01.

**Table 3:** The estimated coefficients of the selected 10 variables on the THEPC and the THEE’s GDP share by the ordinary least squares regression model.

	THEPC (Chinese Yuan)			THEE as a Share of GDP (%)		
	(1)	(2)	(3)	(1)	(2)	(3)
GDPPC	40.187 ** (10.557)	36.752 ** (9.827)	46.462 ** (11.522)	−0.012 * (0.021)	−0.014 * (0.011)	−0.017 ** (0.009)
UR	−42.574 * (11.225)	−39.721 ** (10.101)	−36.136 * (8.199)	−0.089 ** (0.018)	−0.099 * (0.037)	−0.079 * (0.022)
PAR	130.815 * (30.081)	127.183 * (25.013)	117.008 ** (20.100)	0.381 * (0.129)	0.481 * (0.122)	0.218 * (0.102)
ASY	880.835 * (152.745)	890.667 ** (102.051)	900.171 * (99.919)	1.221 ** (0.511)	1.200 * (0.311)	1.100 ** (0.111)
ANHW	36.680 * (9.871)	40.860 * (11.812)	35.963 ** (10.041)	0.061 ** (0.019)	0.050 ** (0.010)	0.072 ** (0.022)
PMAC	18.771 * (5.214)	16. 388 ** (4.547)	19.114 * (6.007)	0.014 * (0.005)	0.028 * (0.006)	0.024 * (0.008)
NDVI	−112.241 * (20.931)	−112.241 * (15.137)	−130.124 ** (16.714)	−0.047 ** (0.009)	−0.039 ** (0.011)	−0.049 ** (0.012)
ASCPC	290.008 * (81.887)	300.811 * (90.122)	310.006 * (80.020)	0.778 * (0.124)	0.801 * (0.106)	0.601 * (0.017)
AVCPC	−30.115 ** (6.679)	−40.617 * (8.688)	−25. 071 * (7.800)	−0.045 ** (0.012)	−0.055 ** (0.009)	−0.039 ** (0.007)
AMCPC	16.186 (17.789)	13.877 (14.781)	18.901 (17.674)	−0.066 (0.071)	−0.076 (0.080)	−0.066 (0.076)
Spatial FE	Control	\	Control	Control	\	Control
Temporal FE	\	Control	Control	\	Control	Control

Footnote: the single * represents a significance of 0.05; the double ** represents a significance of 0.01. FE: fixed effect.

**Table 4:** The B-LASSO model’s estimated regression coefficients of the 10 influence factors on THEPC (Chinese yuan) at subnational scale, east, middle, and west of the Chinese mainland.

	East			Middle			West		
	Mean	2.5%th	97.5%th	Mean	2.5%th	97.5%th	Mean	2.5%th	97.5%th
GDPPC	40.572 **	10.290	78.333	43.282 **	24.438	77.545	58.574 **	27.380	88.472
UR	−19.762	−11.318	59.205	19.726	−51.080	87.293	−9.726	−62.776	41.556
PAR	106.476	−178.762	352.640	169.248 **	120.208	367.907	130.754 *	29.206	291.612
ASY	1269.281 **	245.791	2271.648	12.159	−691.306	720.834	−0.937	−43.682	44.174
ANHW	3.301	−119.395	114.993	−1.329	−59.803	55.018	41.811	−30.075	112.230
PMAC	14.698 *	5.726	18.331	8.369 *	4.107	13.495	8.510	−28.025	9.726
NDVI	146.012	−61.849	915.847	139.608	−52.943	914.759	−97.989 *	−127.624	−37.614
ASCPC	−224.108	−273.397	199.116	−212.123	−129.414	889.737	1.462	−4.907	3.209
AVCPC	−46.062 **	−93.639	−4.325	−13.985	−44.996	14.061	−6.464	−22.149	11.610
AMCPC	−16.424	−176.776	126.207	−14.911	−75.151	42.496	−21.853	−57.364	14.403

Footnote: the single * represents a significance of 0.05; the double ** represents a significance of 0.01.

**Table 5:** The B-LASSO model’s estimated regression coefficients of the 10 influence factors on THEE as a share of GDP (%) at subnational scale, east, middle, and west of the Chinese mainland.

	East			Middle			West		
	Mean	2.5%th	97.5%th	Mean	2.5%th	97.5%th	Mean	2.5%th	97.5%th
GDPPC	−0.090	−0.276	0.097	−0.239	−0.682	0.232	0.003	−0.428	0.488
UR	−0.009	−0.075	0.051	0.059	−0.035	0.168	−0.063 *	−0.150	−0.012
PAR	0.062	−0.107	0.235	0.413 **	0.141	0.673	0.381 **	0.126	0.651
ASY	1.246 **	0.588	1.896	0.012	−0.923	0.965	−0.019	−0.778	0.641
ANHW	−0.001	−0.085	0.070	−0.007	−0.087	0.074	0.082 *	0.072	0.201
PMAC	0.036 **	0.010	0.064	0.022 **	0.008	0.048	0.034	−0.065	0.061
NDVI	0.163	−3.326	7.044	0.345	−6.281	13.489	−0.338 **	−1.559	−0.128
ASCPC	−1.159 *	−2.913	−0.333	−0.337	−1.770	1.272	0.037	−0.613	0.662
AVCPC	−0.040 **	−0.074	−0.005	−0.044 **	−0.088	−0.011	−0.010	−0.037	0.016
AMCPC	−0.069	−0.179	0.033	−0.030	−0.115	0.057	−0.063 **	−0.121	−0.002

Footnote: the single * represents a significance of 0.05; the double ** represents a significance of 0.01.

## Data Availability

All the data in this paper can be accessed in public.
